# Variation in Patient Profiles and Outcomes in US and Non-US Subgroups of the Cangrelor Versus Standard Therapy to Achieve Optimal Management of Platelet Inhibition (CHAMPION) PHOENIX Trial

**DOI:** 10.1161/CIRCINTERVENTIONS.116.003612

**Published:** 2016-06-21

**Authors:** Muthiah Vaduganathan, Robert A. Harrington, Gregg W. Stone, Ph. Gabriel Steg, C. Michael Gibson, Christian W. Hamm, Matthew J. Price, Jayne Prats, Efthymios N. Deliargyris, Kenneth W. Mahaffey, Harvey D. White, Deepak L. Bhatt

**Affiliations:** From the Brigham and Women’s Hospital Heart & Vascular Center and Harvard Medical School, Boston, MA (M.V., D.L.B); Stanford University Medical School, CA (R.A.H., K.W.M.); Columbia University Medical Center and the Cardiovascular Research Foundation, New York City, NY (G.W.S.); FACT (French Alliance for Cardiovascular clinical Trials), DHU FIRE, INSERM Unité 1148, Université Paris-Diderot, and Hôpital Bichat, Assistance-Publique–Hôpitaux de Paris, France, and NHLI, Imperial College, Royal Brompton Hospital, London, UK (P.G.S.); Division of Cardiology, Beth Israel Deaconess Medical Center, Boston, MA (C.M.G.); Kerckhoff Heart and Thorax Center, Bad Nauheim, Germany (C.W.H.); Scripps Clinic and Scripps Translational Science Institute, La Jolla, CA (M.J.P.); The Medicines Company, Parsippany, NJ (J.P., E.N.D.); and Green Lane Cardiovascular Service, Auckland, New Zealand (H.D.W).

**Keywords:** antiplatelet therapy, clinical trial, international comparison, percutaneous coronary intervention, variation

## Abstract

**Background—:**

The Cangrelor Versus Standard Therapy to Achieve Optimal Management of Platelet Inhibition (CHAMPION) PHOENIX trial demonstrated superiority of cangrelor in reducing ischemic events at 48 hours in patients undergoing percutaneous coronary intervention compared with clopidogrel.

**Methods and Results—:**

We analyzed all patients included in the modified intention-to-treat analysis in US (n=4097; 37.4%) and non-US subgroups (n=6845; 62.6%). The US cohort was older, had a higher burden of cardiovascular risk factors, and had more frequently undergone prior cardiovascular procedures. US patients more frequently underwent percutaneous coronary intervention for stable angina (77.9% versus 46.2%). Almost all US patients (99.1%) received clopidogrel loading doses of 600 mg, whereas 40.5% of non-US patients received 300 mg. Bivalirudin was more frequently used in US patients (56.7% versus 2.9%). At 48 hours, rates of the primary composite end point were comparable in the US and non-US cohorts (5.5% versus 5.2%; *P*=0.53). Cangrelor reduced rates of the primary composite end point compared with clopidogrel in US (4.5% versus 6.4%; odds ratio 0.70 [95% confidence interval 0.53–0.92]) and in non-US patients (4.8% versus 5.6%; odds ratio 0.85 [95% confidence interval 0.69–1.05]; interaction *P*=0.26). Similarly, rates of the key secondary end point, stent thrombosis, were reduced by cangrelor in both regions. Rates of Global Use of Strategies to Open Occluded Arteries (GUSTO)–defined severe bleeding were low and not significantly increased by cangrelor in either region.

**Conclusions—:**

Despite broad differences in clinical profiles and indications for percutaneous coronary intervention by region in a large global cardiovascular clinical trial, cangrelor consistently reduced rates of ischemic end points compared with clopidogrel without an excess in severe bleeding in both the US and non-US subgroups.

**Clinical Trial Registration—:**

URL: http://www.clinicaltrials.gov. Unique identifier: NCT01156571.

## Abstract 2

Supplemental Digital Content is available in the text.

**WHAT IS KNOWN**

- Cangrelor is a rapidly acting, potent, reversible intravenous platelet P2Y_12_ receptor antagonist.
- Cangrelor Versus Standard Therapy to Achieve Optimal Management of Platelet Inhibition (CHAMPION) PHOENIX was a global, phase III randomized controlled trial, which formed the basis of cangrelor’s approval for use in patients undergoing percutaneous coronary intervention

**WHAT THE STUDY ADDS**

- Despite substantial international variation in clinical profiles and indications for percutaneous coronary intervention by region, cangrelor consistently reduced rates of 48-hour ischemic complications during percutaneous coronary intervention without a significant excess in severe bleeding compared with clopidogrel in both the US and non-US subgroups of the CHAMPION PHOENIX trial.
- Approximately 40% of patients included in CHAMPION PHOENIX were enrolled from the United States at an enrollment rate that was comparable to non-US sites.
- In an era of decreasing US research engagement, robust US site participation in a global percutaneous coronary intervention trial was feasible with comparable regional results.

Cangrelor is a rapidly acting, potent, reversible intravenous platelet P2Y_12_ adenosine diphosphate receptor antagonist that recently received approval by the Food and Drug Administration for use in patients undergoing percutaneous coronary intervention (PCI).^1^ Cangrelor significantly reduced 48-hour ischemic complications, including stent thrombosis (ST), during PCI without a significant excess in severe bleeding compared with clopidogrel in the Cangrelor Versus Standard Therapy to Achieve Optimal Management of Platelet Inhibition (CHAMPION) PHOENIX trial.^2,3^ These primary results were consistently demonstrated across major subgroups. Despite the lack of any significant interaction in the prespecified treatment-by-region analyses, an in-depth characterization of the geographic variation in clinical profiles and outcomes in this global, contemporary PCI trial is worthwhile. Indeed, differences in event rates between countries have been estimated in some cardiovascular trials to be larger than the observed treatment effects of the study intervention.^4,5^

CHAMPION PHOENIX enrolled 11 145 patients from 153 global sites from 12 different countries, with almost 40% of patients enrolled from the United States.^2^ In spite of application of strict inclusion and exclusion criteria, some variability in the patients enrolled in each geographic region is expected, as observed in recent large antiplatelet trial programs.^6,7^ Geographic heterogeneity in trial outcomes may have important implications in regional approval by regulatory bodies. In this prespecified subgroup analysis, we describe the baseline characteristics, safety and efficacy end points, and response to cangrelor in patients enrolled in US and non-US sites in CHAMPION PHOENIX.

## Methods

### Study Population

The study design,^8^ protocol,^9^ and primary results^2^ of CHAMPION PHOENIX have been described previously. In brief, CHAMPION PHOENIX was an international, prospective, double-blind, double-dummy, active-controlled trial designed to examine the periprocedural safety and efficacy of cangrelor compared with oral clopidogrel administered at the time of PCI. Patients ≥18 years of age requiring PCI for stable angina, non–ST-segment–elevation acute coronary syndromes, or ST-segment–elevation myocardial infarction (MI) were eligible for enrollment. Patients were excluded if they received a P2Y_12_ antagonist or abciximab within 7 days of randomization or a glycoprotein IIb/IIIa inhibitor or fibrinolytic therapy within 12 hours of randomization. The protocol was approved by the institutional review boards or ethics committees at each participating center, and written informed consent was obtained from all enrolled patients.

### Study Treatment

Cangrelor or matching placebo was given as a bolus (30 μg/kg) and infusion (4 μg/kg per minute) during PCI and for 2–4 hours afterward. A clopidogrel loading dose (600 or 300 mg, at the discretion of the operator) or matching placebo was given at the time of PCI. Approximately 2 hours after PCI, the infusion (cangrelor or placebo) was discontinued and then patients received clopidogrel 600 mg (in the cangrelor arm) or matching placebo (in the clopidogrel arm). All patients received aspirin (75–325 mg). Clopidogrel 75 mg was administered during the first 48 hours, after which P2Y_12_ inhibition was left to the discretion of the site investigator. Similarly, selection of access site, stent type, sheath management protocol, and periprocedural anticoagulation were determined by local site investigators. Rescue glycoprotein IIb/IIIa inhibitors were reserved for management of periprocedural thrombotic complications.

### Study End Points

This prespecified subgroup analysis^9^ evaluated the same safety and efficacy end points as the main CHAMPION PHOENIX trial. Consistent with the overall CHAMPION PHOENIX analytic scheme, efficacy end points were assessed in the modified intention-to-treat population, which included all patients who underwent PCI and received study drug. The safety end points were assessed in patients who underwent randomization and received at least one dose of the study drug. The primary efficacy end point was the composite rate of all-cause mortality, MI, ischemia-driven revascularization, or ST at 48 hours after randomization. The key secondary efficacy end point was the incidence of ST at 48 hours, which included both Academic Research Consortium–defined ST^10^ and intraprocedural ST (defined as new or worsened stent-related thrombus as assessed by frame-by-frame analysis by a blinded angiographic core laboratory [Cardiovascular Research Foundation, New York City, NY]).^3^ MI was defined according to the second universal definition.^11^ Secondary efficacy events occurring at 30 days postrandomization, including death, MI, ischemia-driven revascularization, or ST, were all specifically adjudicated by an independent and blinded Clinical Events Committee (Duke Clinical Research Institute, Durham, NC). The primary safety end point was noncoronary artery bypass graft–related severe/life-threatening bleeding, according to Global Use of Strategies to Open Occluded Arteries (GUSTO) criteria at 48 hours. Requirement for transfusion and other bleeding indices including Thrombolysis in Myocardial Infarction (TIMI) and Acute Catheterization and Urgent Intervention Triage Strategy (ACUITY) were also assessed.

### Statistical Analysis

All primary and secondary analyses detailed in the main trial publication^2^ were repeated separately in the US versus the non-US subgroups, as designated by the original trial protocol.^9^ Efficacy and safety end points were compared between geographic regions and between cangrelor and clopidogrel arms within each individual region. Logistic regression analyses were used to estimate effect sizes, expressed as odds ratios (OR) and 95% confidence intervals (CI). To account for minor discrepancies in clinical profiles between treatment arms in US and non-US subsets, multiple logistic regression models were adjusted for covariates which differed between treatment arms (*P*<0.15) in either regional subset. Treatment-by-region interaction analyses for each of the safety and efficacy end points were tested using the Breslow–Day method. Enrollment rates per country and region were calculated and expressed as number of patients per site per month with enrollment duration estimated from study start and end dates. Continuous variables are presented as mean±standard deviation (SD) or as median (interquartile range [Q1, Q3]) and compared using Student’s *t* tests or Wilcoxon rank-sum tests, as appropriate. Categorical variables are presented as n (%) and compared using chi-squared testing or Fisher’s exact tests, as appropriate. Kaplan–Meier curves by region were constructed for the primary efficacy and safety end points and key secondary end point and compared using log-rank tests. No adjustments were made for multiple comparisons. All statistical analyses were performed using SAS software, version 9.3 (SAS Institute, Cary, NC).

## Results

**Figure 1.**
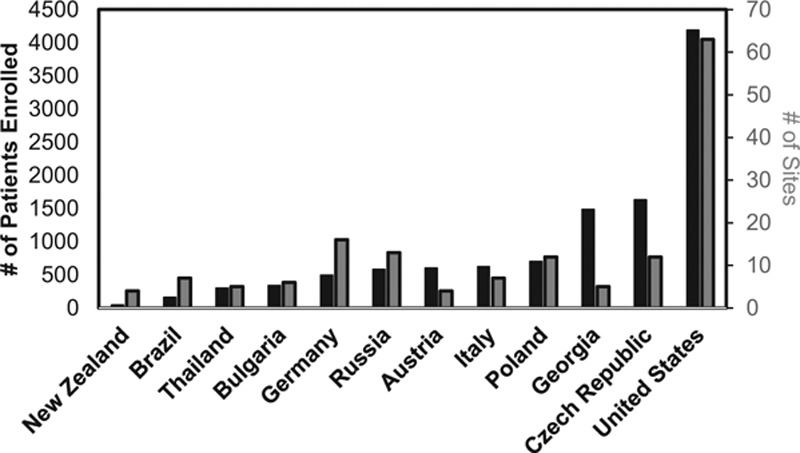
Number of enrolled patients (black bars) and sites (gray bars) per country in the Cangrelor Versus Standard Therapy to Achieve Optimal Management of Platelet Inhibition (CHAMPION) PHOENIX trial.

From September 30, 2010 to October 3, 2012, CHAMPION PHOENIX randomized 11 145 patients enrolled from 153 global sites from 12 countries (Austria, Brazil, Bulgaria, Czech Republic, Georgia, Germany, Italy, New Zealand, Poland, Russia, Thailand, and the United States). The number of enrolled subjects and sites per country varied (Figure 1). The United States enrolled the highest number of patients (n=4188; 37.6%) from 63 enrolling sites. Of this randomized cohort, 10 942 patients (98.2%) ultimately underwent PCI and received the assigned drug and were included in the intention-to-treat analysis. Follow-up was available at 48 hours and 30 days in 10 939 and 10 919 patients, respectively. The final analytic cohort was based on the intention-to-treat trial population, and the present analysis compared the clinical profiles of patients enrolled from the US (n=4097; 37.4%) versus non-US sites (n=6845; 62.6%).

### Baseline Characteristics

Baseline characteristics were well-balanced between cangrelor and clopidogrel arms within each region (Table I in the Data Supplement). However, distinct differences in demographic, clinical, and angiographic characteristics were observed in almost every variable between US and non-US regions (Table 1). The US cohort was older and more likely to be female (*P*<0.001 for both). Over 90% of CHAMPION PHOENIX participants were white, regardless of region, but the US group had higher rates of black and Hispanic/Latino participation (*P*<0.001). US patients consistently had higher rates of comorbid diseases (including diabetes mellitus, hypertension, hyperlipidemia, peripheral artery disease, heart failure), prior PCI or coronary artery bypass graft, and family history of coronary artery disease (all comparisons, *P*<0.001). Stable angina was more frequently the indication for PCI in US compared with non-US patients (77.9% versus 46.2%), whereas non–ST-segment–elevation acute coronary syndromes (19.2% versus 30.8%) and ST-segment–elevation MI (2.9% versus 23.0%) were more common indications outside the United States (*P*<0.001). Cardiac biomarkers were abnormal at baseline in 45.2% in the non-US subgroup compared with 21.7% in the US subgroup (*P*<0.001). Regional variation was also observed in periprocedural medication administration. Almost all US patients (99.1%) were intended to receive clopidogrel loading doses of 600 mg, whereas 40.5% of non-US patients were intended to receive 300 mg (*P*<0.001). Bivalirudin was more frequently used in US patients (56.7% versus 2.9%), whereas other anticoagulants were used more frequently in non-US patients (all comparisons, *P*<0.001). Radial access (29.8% versus 23.9%) and drug-eluting stents (68.8% versus 47.7%) were used at higher rates in the US cohort versus non-US cohort (*P*<0.001 for both).

**Table 1.**
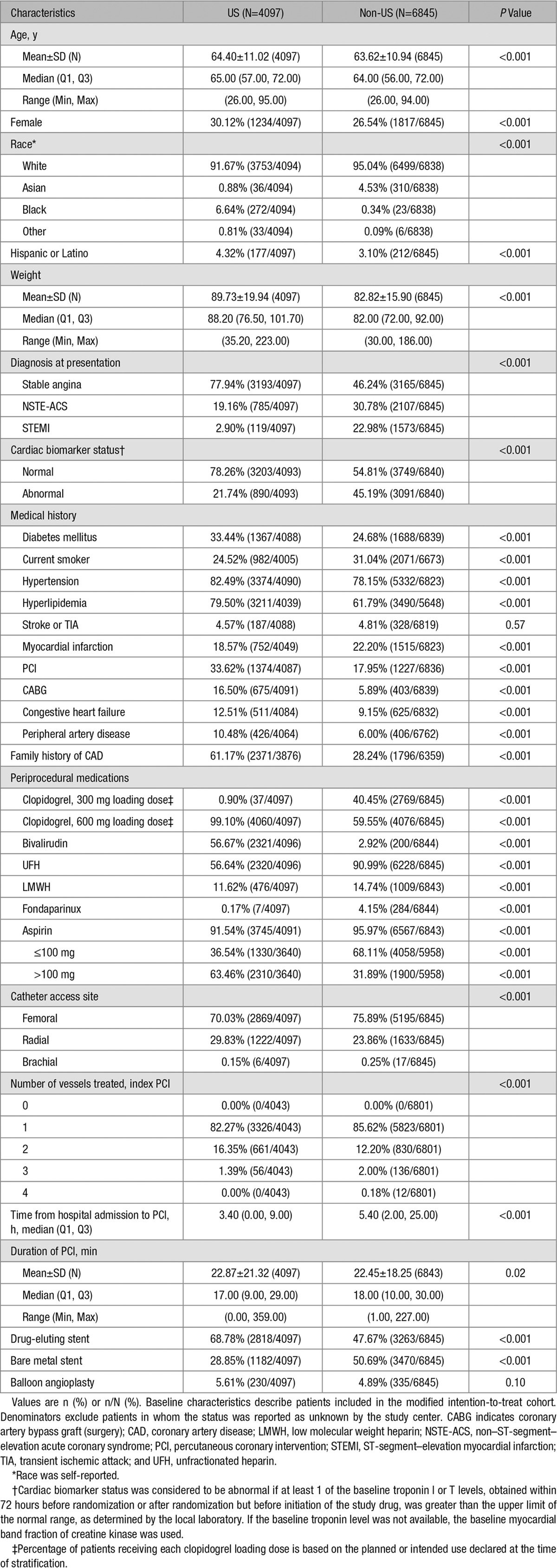
Baseline Characteristics in US and Non-US Subgroups

### Regional Enrollment Rates

Enrollment rates did not differ substantially between US sites (2.7 patients/site per month) and non-US sites (3.2 patients/site per month). However, enrollment rates ranged markedly across countries from 0.5 patients/site per month in New Zealand to 12.1 patients/site per month in Georgia.

### Primary Efficacy End Point

**Figure 2.**
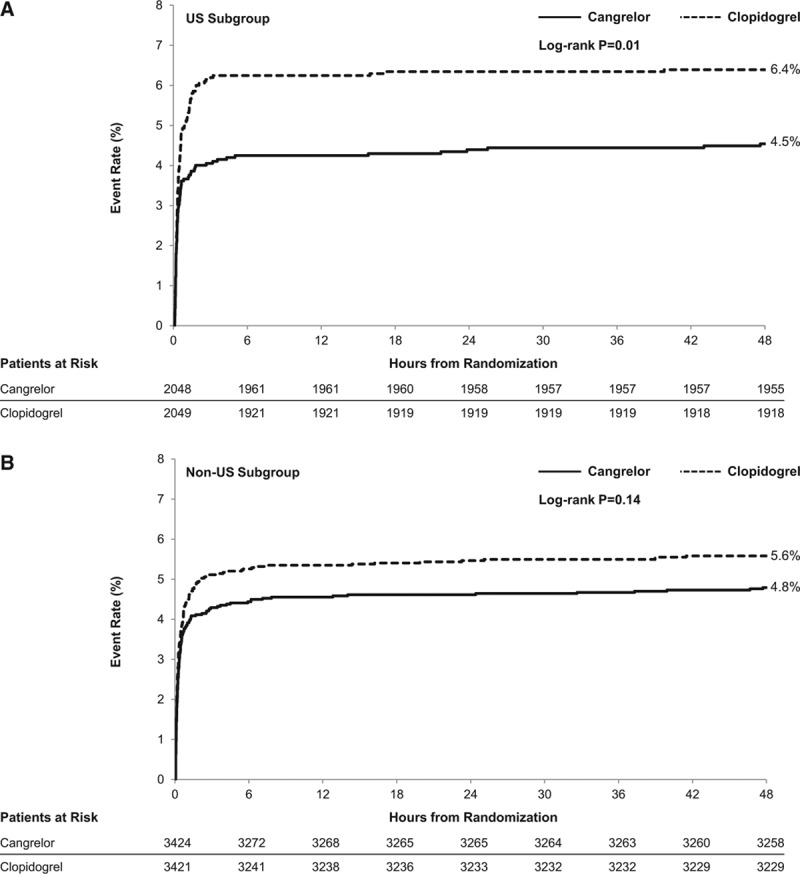
Kaplan–Meier failure curves for the primary efficacy end point in US (**A**) and non-US (**B**) subgroups. The primary efficacy end point of composite of death from any cause, myocardial infarction, ischemia-driven revascularization, or stent thrombosis at 48 hours after randomization was reduced by cangrelor in both US and non-US subgroups (interaction *P*=0.26) compared with clopidogrel in the modified intention-to-treat population (which comprised patients who underwent percutaneous coronary intervention and received the study drug). Failure functions were compared by region using the log-rank test.

The main outcomes are displayed by region and treatment assignment in Table 2. A total of 224 patients (5.5%) in the US cohort and 355 patients (5.2%) in the non-US cohort experienced the primary composite efficacy end point of death from any cause, MI, ischemia-driven revascularization, or ST at 48 hours (*P*=0.53). Rates of the primary composite end point were lower in the cangrelor arm compared with the clopidogrel arm in US (4.5% versus 6.4%; OR 0.70 [95% CI 0.53–0.92]) and non-US patients (4.8% versus 5.6%; OR 0.85 [95% CI 0.69–1.05]); interaction *P*=0.26. Multiple logistic regression analyses accounted for age, body weight, cardiac biomarker status, current smoking status, prior MI, previous coronary artery bypass graft, and history of peripheral artery disease. Even after accounting for the minimal observed variation between treatment arms in regional subgroups, cangrelor consistently reduced the primary end point compared with clopidogrel in US (adjusted OR 0.69 [95% CI 0.52–0.91]) and non-US subsets (adjusted OR 0.82 [95% CI 0.66–1.03]); adjusted interaction *P*=0.34. Figure I in the Data Supplement displays comparative unadjusted OR estimates for each individual country. Kaplan–Meier estimates of the time-to-primary end point are shown in the US (Figure 2A) and non-US subgroups (Figure 2B).

**Table 2.**
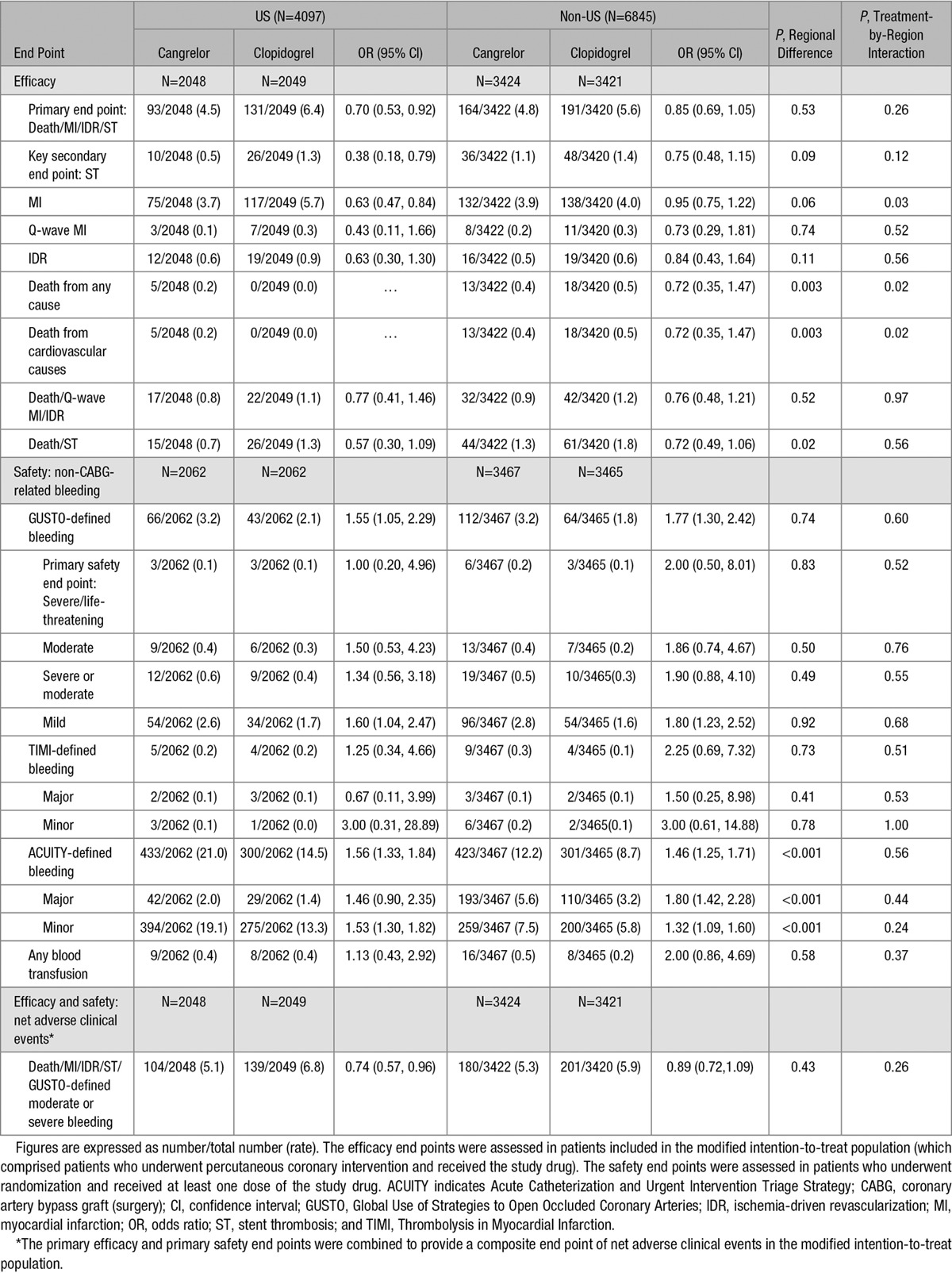
Efficacy and Safety End Points at 48 h After Randomization in US and Non-US Subgroups

### Stent Thrombosis

Similarly, 36 patients (0.9%) in the United States and 84 patients (1.2%) outside the United States experienced ST at 48 hours (*P*=0.09). Cangrelor reduced rates of ST in patients enrolled in the United States (0.5% versus 1.3%; OR 0.38 [95% CI 0.18–0.79]) and outside the United States (1.1% versus 1.4%; OR 0.75 [95% CI 0.48–1.15]); interaction *P*=0.12 (Figure II in the Data Supplement).

### Other Efficacy End Points

MI was the most frequent efficacy outcome and accounted for 85.7% and 76.1% of the primary composite events in the US and non-US cohorts, respectively. Cangrelor significantly reduced risk of MI in US patients (3.7% versus 5.7%; *P*=0.002), but not in non-US patients (3.9% versus 4.0%; *P*=0.71) compared with clopidogrel (interaction *P*=0.03). This geographic heterogeneity of treatment effect on MI extended to 30 days (Table II in the Data Supplement). All-cause mortality rates at 48 hours were low with very few events worldwide (0.1% in US patients and 0.5% in non-US patients). The need for rescue glycoprotein IIb/IIIa inhibitors was lower in patients assigned to cangrelor compared with clopidogrel in both regions. Other secondary efficacy end points by region and treatment assignment are described in detail in Table II in the Data Supplement.

### Safety End Points

The rates of the primary safety end point, GUSTO-defined severe/life-threatening bleeding, were low in both US (0.15%) and non-US regions (0.13%; Table 2). There was no significant treatment heterogeneity in cangrelor effect between regions in the primary safety end point (Figure III in the Data Supplement), TIMI-defined bleeding, ACUITY-defined bleeding, or the need for blood transfusions.

In post hoc analysis, the primary efficacy and safety end points were combined to provide a composite end point of net adverse clinical events, which was reduced in the US by cangrelor (5.1% versus 6.8%) and in non-US regions (5.3% versus 5.9%); interaction *P*=0.26.

## Discussion

Consistent with the overall CHAMPION PHOENIX results, this prespecified analysis confirmed relative homogeneity in the primary efficacy and safety end points for cangrelor versus clopidogrel in US and non-US subsets undergoing elective or urgent PCI. Furthermore, cangrelor consistently reduced net adverse clinical events (the composite of the primary efficacy and safety end points) compared with clopidogrel, regardless of geographic region. Challenges in site-based enrollment, economic pressures to complete trial protocols on shorter timelines, and improvements in background drug and device therapies have driven recent patterns of globalization of cardiovascular clinical trials.^12^ These secular trends have presented unique challenges and opportunities to dissect differential treatment responses by region in large global cardiovascular clinical trials. Unfortunately, fewer US sites are participating in emerging cardiovascular mega-trials. For instance, only 8% of all randomized patients in the Platelet Inhibition and Patient Outcomes (PLATO) trial were enrolled from US sites.^6^

In the context of declining US clinical trial participation, US share in global research funding,^13^ and US growth in Food and Drug Administration–regulated investigators,^14^ CHAMPION PHOENIX clarifies that US engagement in global clinical trials in PCI is feasible. US sites enrolled the highest proportion (38%) of randomized patients in CHAMPION PHOENIX from only 63 sites. Furthermore, efficiency of enrollment was preserved with comparable enrollment rates across global regions. Site-based enrollment practices may shape patterns of patient profiles, protocol completion, and trial outcomes, potentially related to variation in the stringency of application of inclusion and exclusion criteria. In the Efficacy of Vasopressin Antagonism in Heart Failure: Outcome Study with Tolvaptan (EVEREST) trial, participants enrolled from low-enrolling sites were independently at higher risk for adverse events.^5,15^ It is thus reassuring that US sites in CHAMPION PHOENIX enrolled a high volume of patients expeditiously comparable to non-US sites (≈3 patients per site per month). This also suggests that the CHAMPION PHOENIX protocol fit nicely into contemporary US practice at many sites.

Regional consistency of primary safety and efficacy end points in CHAMPION PHOENIX may be potentially related to several factors. First, despite wide differences in clinical profiles and PCI indications, overall event rates in US and non-US cohorts were similar. This international event burden may reflect standardization of global cardiovascular practices, guidelines, and background therapies. Rates of postprocedural MI have been reported to be higher in US compared with non-US sites in certain experiences,^16,17^ but may have been offset in CHAMPION PHOENIX because of differential case mix, with a greater proportion of nonelective PCI performed in non-US sites. Second, all enrolling sites were PCI-capable and thus may possess certain unifying characteristics. Third, specific adjudication of end points by an independent Clinical Events Committee and a blinded angiographic core laboratory may minimize regional variability in end point assessment and treatment effects. Finally, its predictable pharmacological profile, near-complete antiplatelet inhibition, and excellent bioavailability may have contributed to the consistency of cangrelor’s actions across various populations of patients.^18^

Minimal heterogeneity is expected around the overall trial point estimate, especially in PCI trials, given systematic differences in indications for PCI and concomitant treatment strategies (left to the discretion of local site investigators). The overall interaction terms were not significant for the primary and secondary efficacy end points, and the directionality of effects (favoring cangrelor) were preserved across regions; as such, the effect sizes and specific point estimates within each regional subset should be regarded with caution. Point estimates for the primary efficacy end point varied substantially by country of enrollment and country-specific sample size, highlighting that small regional experiences may provide unstable estimates. Geographic variation in secondary treatment outcomes may arise from variability in patient-related factors, regional medical practice, and end point assessment.^19^ Despite adherence to strict criteria of enrollment, patients enrolled from different regions may vary in important ways, which may in turn influence treatment risk–benefit ratios, side effect profiles, and adherence patterns. US participants in CHAMPION PHOENIX had higher rates of established cardiovascular disease and cardiovascular risk factors, and over 70% underwent PCI for stable angina. In contrast with the findings related to the primary safety and efficacy end points, the risk reduction of periprocedural MI by cangrelor appeared to be confined to the US subgroup, which may be explained by several potential factors. Despite the use of the standardized, universal definition of post-PCI MI (type 4a)^11^ that leveraged adjunctive evidence of ischemia based on symptom reporting, angiography, and electrocardiography and an independent angiographic core laboratory for ST (type 4b),^3,10^ higher rates of PCI for acute coronary syndrome in non-US participants may have confounded the detection of periprocedural MI. Historically, nonfatal ischemic end points, such as MI, have been subject to underreporting and potentially greater regional influence. In CHAMPION PHOENIX, however, post-PCI biomarkers were collected per protocol and processed in core laboratories for almost all adjudicated MIs. Processing of biomarker samples in local laboratories was only required if these were not available, and utilization of local laboratories did not differ by region (US versus non-US).

Review of region-specific trial data may influence the regulatory approval process. The Food and Drug Administration is increasingly requesting pivotal clinical trials to include a certain proportion of patients enrolled from the United States. Regulatory bodies need to ensure representativeness and consistency of efficacy and safety between the US subgroup and the overall trial sample. Subgroup analyses of the PLATO trial revealed that patients in North America assigned to ticagrelor experienced a higher rate of the primary end point of cardiovascular death, MI, or stroke compared with clopidogrel; this treatment effect was disparate from that observed in other geographic regions.^6^ Three independent analytic teams have come to the conclusion that differences in aspirin maintenance dose may partially explain these regional discrepancies in ticagrelor efficacy.^6^ This PLATO analysis has prompted the Food and Drug Administration to issue a black-box warning against the use of ticagrelor with aspirin doses exceeding 100 mg/d. Thus, region-specific data from emerging cardiovascular trial programs may be important for regulatory approval, specific labeling, and restriction of use.

Despite the negative treatment-by-region interaction for the primary end points, it is still worthwhile to report and analyze regional data emerging from contemporary cardiovascular clinical trials. These trial programs present unique opportunities to describe evolving patient profiles, treatment practices, and cardiovascular disease burden in a well-monitored global context. These region-specific data should be carefully interpreted to identify true geographic heterogeneity.

There are several limitations to this prespecified subgroup analysis. The overall trial was not powered to assess treatment effects by region. We did not adjust for multiple testing, and thus, heterogeneity across secondary end points may be due to chance alone from multiplicity of testing. The comparison of treatment effects in US and non-US subgroups was prespecified in this trial, and thus, we did not dissect the trial cohort further by specific region, country, or site. As such, we combined regional data from all non-US sites, which may not be entirely uniform. Newer P2Y_12_ receptor antagonists, such as prasugrel and ticagrelor, were not used in this trial.

CHAMPION PHOENIX demonstrated reduced rates of 48-hour ischemic events with cangrelor compared with clopidogrel in patients undergoing PCI irrespective of region, without any excess in severe bleeding complications or transfusions. Important differences in demographic and clinical characteristics, background therapies, and interventional factors were observed across the world. Despite this substantial international variation in clinical profiles and indications for PCI, treatment effects on the primary safety and efficacy end points did not differ significantly by geographic region. Given increasing globalization of cardiovascular clinical trial programs, clinical trialists, regulatory authorities, and sponsors should continue to monitor and evaluate for potential regional variation in drug/device safety or efficacy. Standardization of end point assessment and selection of high-quality sites may minimize regional heterogeneity. In an era of decreasing US research engagement, robust US site participation in a global PCI trial was feasible with comparable regional results.

## Acknowledgments

We thank Steven E. Elkin, MS, and Debra Bernstein, PhD, of The Medicines Company for their statistical support, along with Yuyin Liu, MS, and Lanyu Lei, MS, of the Harvard Clinical Research Institute for their independent verification of the analyses. Harvard Clinical Research Institute received funding from The Medicines Company for these analyses.

## Disclosures

The CHAMPION-PHOENIX trial was funded by The Medicines Company. Dr Harrington discloses the following relationships—Advisory Board: Evidint, Regado, Scanadu; Honoraria: Amgen, Daiichi-Lilly, Gilead Sciences Inc, Janssen R&D, Medtronic, Merck, Novartis Corporation, The Medicines Company, Vida Health, Vox Media, WebMD; Other: American Heart Association; Research Funding: AstraZeneca, Bristol-Myers Squibb, CSL Behring, GSK, Merck, Portola, Sanofi-aventis, The Medicines Company; Ownership Interest: Element Science, MyoKardia. Dr Steg discloses the following relationships—Research Funding (to INSERM U1148): Sanofi, Servier; Speaking or Consultant Fees: Amarin, AstraZeneca, Bayer, Boehringer-Ingelheim, Bristol-Myers Squibb, CSL-Behring, Daiichi-Sankyo, GlaxoSmithKline, Janssen, Lilly, Novartis, Pfizer, Regeneron, Roche, Sanofi, Servier, The Medicines Company; Stock Ownership: Aterovax. Dr Gibson discloses the following relationships—Honoraria: The Medicines Company. Dr Hamm discloses the following relationships—Honoraria: AstraZeneca, Sanofi Aventis, Lilly; Research Funding: Astra Zeneca, The Medicines Company. Dr Price discloses the following relationships—Honoraria: AstraZeneca, Merck & Co, Accriva Diagnostics, The Medicines Company. Dr Prats discloses the following relationships—Employment: The Medicines Company. Dr Deliargyris discloses the following relationships—Employment: The Medicines Company. Dr Mahaffey discloses the following relationships—Honoraria: Bayer, Boehringer Ingelheim, Bristol-Myers Squibb, Cubist, Eli Lilly, Epson, Forest, Glaxo Smith Kline, Johnson & Johnson, Medtronic, Merck, Mt. Sinai, Myokardia, Omthera, Portola, Purdue Pharma, Spring Publishing, Vindico, WebMD; Research Funding: Daiichi, Johnson & Johnson, Medtronic, St Jude, Tenax. Dr White discloses the following relationships—Honoraria: AstraZeneca; Research Funding: Sanofi-Aventis, Eli Lilly, National Health Institute, Glaxo Smith Kline, Merck Sharpe & Dohme, AstraZeneca. Dr Bhatt discloses the following relationships—Advisory Board: Cardax, Elsevier Practice Update Cardiology, Medscape Cardiology, Regado Biosciences; Board of Directors: Boston VA Research Institute, Society of Cardiovascular Patient Care; Chair: American Heart Association Quality Oversight Committee; Data Monitoring Committees: Duke Clinical Research Institute, Harvard Clinical Research Institute, Mayo Clinic, Population Health Research Institute; Honoraria: American College of Cardiology (Senior Associate Editor, Clinical Trials and News, ACC.org), Belvoir Publications (Editor in Chief, Harvard Heart Letter), Duke Clinical Research Institute (clinical trial steering committees), Harvard Clinical Research Institute (clinical trial steering committee), HMP Communications (Editor in Chief, Journal of Invasive Cardiology), Journal of the American College of Cardiology (Guest Editor; Associate Editor), Population Health Research Institute (clinical trial steering committee), Slack Publications (Chief Medical Editor, Cardiology Today’s Intervention), Society of Cardiovascular Patient Care (Secretary/Treasurer), WebMD (CME steering committees); Other: Clinical Cardiology (Deputy Editor), NCDR-ACTION Registry Steering Committee (Vice-Chair), VA CART Research and Publications Committee (Chair); Research Funding: Amarin, AstraZeneca, Bristol-Myers Squibb, Eisai, Ethicon, Forest Laboratories, Ischemix, Medtronic, Pfizer, Roche, Sanofi Aventis, The Medicines Company (including for his role as Co-Chair of CHAMPION PHOENIX); Royalties: Elsevier (Editor, Cardiovascular Intervention: A Companion to Braunwald’s Heart Disease); Site Co-Investigator: Biotronik, Boston Scientific, St Jude Medical; Trustee: American College of Cardiology; Unfunded Research: FlowCo, PLx Pharma, Takeda. All other authors have reported that they have no other relationships relevant to the contents of this article to disclose.

## Supplementary Material
