# Supplementary material for: Variation in Patient Profiles and Outcomes in US and Non-US Subgroups of the Cangrelor Versus Standard Therapy to Achieve Optimal Management of Platelet Inhibition (CHAMPION) PHOENIX Trial
Source: Circ Cardiovasc Interv. 2016 Jun 21;9(6):e003612. doi: 10.1161/CIRCINTERVENTIONS.116.003612 (PMC4920208; doi:10.1161/CIRCINTERVENTIONS.116.003612)
Supplement: Supplementary file 1 [file hcv-9-e003612-s001.pdf]

SUPPLEMENTAL MATERIAL

These tables and figures are intended for publication as an online data supplement.

Supplemental Table 1. Baseline Characteristics by Treatment Group in US and non-US subgroups

Characteristics	US Cangrelor (N=2048)	US Clopidogrel (N=2049)	US p-value	Non-US Cangrelor (N=3424)	Non-US Clopidogrel (N=3421)	Non-US p-value
Age, yrs						
Mean±SD (N)	64.71±11.07 (2048)	64.09±10.96 (2049)	0.07	63.66±10.89 (3424)	63.58±10.99 (3421)	0.77
Median (Q1, Q3)	65.00 (57.00,73.00)	64.00 (56.00,72.00)		63.00 (56.00,72.00)	64.00 (56.00,72.00)	
Range (Min, Max)	(26.00,91.00)	(27.00,95.00)		(28.00,94.00)	(26.00,92.00)	
Female	30.66% (628/2048)	29.58% (606/2049)	0.45	27.16% (930/3424)	25.93% (887/3421)	0.25
Race*			0.55			0.86
White	91.84% (1880/2047)	91.50% (1873/2047)		95.03% (3252/3422)	95.05% (3247/3416)	
Asian	0.68% (14/2047)	1.07% (22/2047)		4.59% (157/3422)	4.48% (153/3416)	
Black	6.74% (138/2047)	6.55% (134/2047)		0.32% (11/3422)	0.35% (12/3416)	
Other	0.73% (15/2047)	0.88% (18/2047)		0.06% (2/3422)	0.12% (4/3416)	
Hispanic or Latino	4.15% (85/2048)	4.49% (92/2049)	0.59	3.15% (108/3424)	3.04% (104/3421)	0.79
Weight						
Mean±SD (N)	89.22±19.67 (2048)	90.23±20.20 (2049)	0.11	82.78±16.04 (3424)	82.86±15.76 (3421)	0.85
Median (Q1, Q3)	88.00 (75.00,101.00)	88.50 (77.10,102.00)		82.00 (72.00,93.00)	82.00 (72.00,92.00)	
Range (Min, Max)	(35.20,186.00)	(38.70,223.00)		(39.00,186.00)	(30.00,155.00)	
Diagnosis at presentation			0.83			0.30
Stable angina	78.32% (1604/2048)	77.55% (1589/2049)		46.20% (1582/3424)	46.27% (1583/3421)	
NSTE-ACS	18.85% (386/2048)	19.47% (399/2049)		31.48% (1078/3424)	30.08% (1029/3421)	
STEMI	2.83% (58/2048)	2.98% (61/2049)		22.31% (764/3424)	23.65% (809/3421)	
Cardiac biomarker status†			0.07			0.33
Normal	79.42% (1625/2046)	77.09% (1578/2047)		55.39% (1895/3421)	54.23% (1854/3419)	
Abnormal	20.58% (421/2046)	22.91% (469/2047)		44.61% (1526/3421)	45.77% (1565/3419)	
Medical history						
Diabetes mellitus	32.60% (666/2043)	34.28% (701/2045)	0.26	24.93% (853/3421)	24.43% (835/3418)	0.63
Current smoker	23.50% (470/2000)	25.54% (512/2005)	0.13	30.97% (1034/3339)	31.10% (1037/3334)	0.90
Hypertension	82.49% (1686/2044)	82.50% (1688/2046)	0.99	78.71% (2688/3415)	77.58% (2644/3408)	0.26
Hyperlipidemia	80.04% (1616/2019)	78.96% (1595/2020)	0.40	61.69% (1747/2832)	61.90% (1743/2816)	0.87
Stroke or TIA	4.99% (102/2043)	4.16% (85/2045)	0.20	4.95% (169/3412)	4.67% (159/3407)	0.58
Myocardial infarction	17.31% (351/2028)	19.84% (401/2021)	0.04	21.71% (741/3413)	22.70% (774/3410)	0.33
PCI	32.79% (670/2043)	34.44% (704/2044)	0.27	17.49% (598/3419)	18.41% (629/3417)	0.32
CABG	17.20% (352/2046)	15.79% (323/2045)	0.23	6.61% (226/3420)	5.18% (177/3419)	0.01
Congestive heart failure	12.10% (247/2041)	12.92% (264/2043)	0.43	8.92% (305/3419)	9.38% (320/3413)	0.51

	US			Non-US		
**Characteristics**	**Cangrelor (N=2048)**	**Clopidogrel (N=2049)**	**p-value**	**Cangrelor (N=3424)**	**Clopidogrel (N=3421)**	**p-value**
Peripheral artery disease	11.48% (233/2030)	9.49% (193/2034)	0.04	6.34% (214/3377)	5.67% (192/3385)	0.25
Family history of CAD	60.06% (1167/1943)	62.29% (1204/1933)	0.16	28.99% (921/3177)	27.50% (875/3182)	0.19
Peri-procedural medications			0.87			0.97
Clopidogrel, 300mg loading dose	0.93% (19/2048)	0.88% (18/2049)		40.48% (1386/3424)	40.43% (1383/3421)	
Clopidogrel, 600mg loading dose	99.07% (2029/2048)	99.12% (2031/2049)		59.52% (2038/3424)	59.57% (2038/3421)	
Bivalirudin	56.59% (1159/2048)	56.74% (1162/2048)	0.93	2.72% (93/3424)	3.13% (107/3420)	0.31
UFH	55.81% (1143/2048)	57.47% (1177/2048)	0.28	91.38% (3129/3424)	90.59% (3099/3421)	0.25
LMWH	11.67% (239/2048)	11.57% (237/2049)	0.92	14.40% (493/3424)	15.09% (516/3419)	0.42
Fondaparinux	0.15% (3/2048)	0.20% (4/2049)	0.71	4.47% (153/3423)	3.83% (131/3421)	0.18
Aspirin	91.55% (1874/2047)	91.54% (1871/2044)	0.99	96.14% (3290/3422)	95.79% (3277/3421)	0.46
≤ 100 mg	36.84% (673/1827)	36.24% (657/1813)	0.71	68.67% (2036/2965)	67.56% (2022/2993)	0.36
> 100 mg	63.16% (1154/1827)	63.76% (1156/1813)		31.33% (929/2965)	32.44% (971/2993)	
Catheter Access Site			0.15			0.96
Femoral	70.85% (1451/2048)	69.20% (1418/2049)		75.99% (2602/3424)	75.80% (2593/3421)	
Radial	29.10% (596/2048)	30.55% (626/2049)		23.77% (814/3424)	23.94% (819/3421)	
Brachial	0.05% (1/2048)	0.24% (5/2049)		0.23% (8/3424)	0.26% (9/3421)	
Number of vessels treated, index PCI			0.54			0.22
0	0.00% (0/2021)	0.00% (0/2022)		0.00% (0/3402)	0.00% (0/3399)	
1	82.24% (1662/2021)	82.29% (1664/2022)		84.74% (2883/3402)	86.50% (2940/3399)	
2	16.18% (327/2021)	16.52% (334/2022)		12.96% (441/3402)	11.44% (389/3399)	
3	1.58% (32/2021)	1.19% (24/2022)		2.09% (71/3402)	1.91% (65/3399)	
4	0.00% (0/2021)	0.00% (0/2022)		0.21% (7/3402)	0.15% (5/3399)	
Duration of PCI, min						
Mean±SD (N)	22.74±21.48 (2048)	23.00±21.16 (2049)	0.96	22.68±18.52 (3423)	22.22±17.98 (3420)	0.21
Median (Q1, Q3)	17.00 (9.00,29.00)	17.00 (9.00,30.00)		18.00 (10.00,30.00)	17.00 (10.00,30.00)	
Range (Min, Max)	(0.00,359.00)	(1.00,205.00)		(1.00,227.00)	(1.00,182.00)	
Time from hospital admission to PCI, hr, median (Q1, Q3)	3.30 (0.00,9.00)	3.40 (0.00,9.00)	0.75	5.40 (2.00,25.00)	5.30 (2.00,24.00)	0.26
Drug-eluting stent	68.99% (1413/2048)	68.57% (1405/2049)	0.77	48.13% (1648/3424)	47.21% (1615/3421)	0.45
Bare metal stent	28.61% (586/2048)	29.09% (596/2049)	0.74	50.29% (1722/3424)	51.10% (1748/3421)	0.51
Balloon angioplasty	5.91% (121/2048)	5.32% (109/2049)	0.41	4.99% (171/3424)	4.79% (164/3421)	0.70

Values are n (%) or n/N (%). Baseline characteristics describe patients included in the modified intention-to-treat cohort. Denominators exclude patients in whom the status was reported as unknown by the study center.

*Race was self-reported.

†Cardiac biomarker status was considered to be abnormal if at least 1 of the baseline troponin I or T levels, obtained within 72 h before randomization or after randomization but before initiation of the study drug, was greater than the upper limit of the normal range, as determined by the local laboratory. If the baseline troponin level was not available, the baseline myocardial band fraction of creatine kinase was used.

Abbreviations: CABG = coronary artery bypass graft (surgery); CAD = coronary artery disease; IQR = interquartile range; LMWH = low molecular weight heparin; NSTE-ACS = non–ST-segment elevation acute coronary syndrome; PCI = percutaneous coronary intervention; STEMI = ST-segment-elevation myocardial infarction; TIA = transient ischemic attack; UF = unfractionated heparin.

Supplemental Table 2. Additional Secondary Efficacy Endpoints at 48 Hours and 30-days in US and non-US Subgroups

	US (N=4,097)			Non-US (N=6,845)				
	Cangrelor	Clopidogrel		Cangrelor	Clopidogrel			
	(N=2,048)	(N=2,049)	OR (95% CI)	(N=3,424)	(N=3,421)	OR (95% CI)	P, Regional Difference	P, Treatment-by-Region Interaction
**48-Hour Post-Randomization**								
**Intra-procedural ST**	8/ 2048(0.4)	16/ 2049(0.8)	0.50(0.21,1.17)	27/ 3422(0.8)	38/ 3420(1.1)	0.71(0.43,1.16)	0.04	0.48
**Definite ST**	3/ 2048(0.1)	11/ 2049(0.5)	0.27(0.08,0.98)	9/ 3422(0.3)	11/ 3420(0.3)	0.82(0.34,1.97)	0.65	0.16
**ARC ST**	3/ 2048(0.1)	11/ 2049(0.5)	0.27(0.08,0.98)	9/ 3422(0.3)	11/ 3420(0.3)	0.82(0.34,1.97)	0.65	0.16
**IDR**	12/ 2048(0.6)	19/ 2049(0.9)	0.63(0.30,1.30)	16/ 3422(0.5)	19/ 3420(0.6)	0.84(0.43,1.64)	0.11	0.56
**PCI**	6/ 2048(0.3)	15/ 2049(0.7)	0.40(0.15,1.03)	15/ 3422(0.4)	18/ 3420(0.5)	0.83(0.42,1.65)	0.83	0.21
**CABG**	6/ 2048(0.3)	4/ 2049(0.2)	1.50(0.42,5.33)	1/ 3422(0.0)	1/ 3420(0.0)	1.00(0.06,15.98)	0.00	0.79
**Death/MI/IDR/ARC-ST**	86/ 2048(4.2)	122/ 2049(6.0)	0.69(0.52,0.92)	144/ 3422(4.2)	164/ 3420(4.8)	0.87(0.69,1.10)	0.17	0.21
**Death/Q-wave MI/IDR/ST**	24/ 2048(1.2)	37/ 2049(1.8)	0.64(0.38,1.08)	56/ 3422(1.6)	75/ 3420(2.2)	0.74(0.52,1.05)	0.10	0.66
**Death/MI/IDR**	86/ 2048(4.2)	122/ 2049(6.0)	0.69(0.52,0.92)	144/ 3422(4.2)	164/ 3420(4.8)	0.87(0.69,1.10)	0.17	0.21
**Death/MI/ST**	87/ 2048(4.2)	127/ 2049(6.2)	0.67(0.51,0.89)	162/ 3422(4.7)	185/ 3420(5.4)	0.87(0.70,1.08)	0.73	0.15
**Death/MI/ARC-ST**	80/ 2048(3.9)	118/ 2049(5.8)	0.67(0.50,0.89)	142/ 3422(4.1)	158/ 3420(4.6)	0.89(0.71,1.13)	0.28	0.12
**Death/Q-wave MI/ST**	17/ 2048(0.8)	30/ 2049(1.5)	0.56(0.31,1.02)	50/ 3422(1.5)	67/ 3420(2.0)	0.74(0.51,1.07)	0.02	0.44
**Death/MI**	79/ 2048(3.9)	117/ 2049(5.7)	0.66(0.49,0.89)	141/ 3422(4.1)	155/ 3420(4.5)	0.91(0.72,1.14)	0.26	0.10
**Death/Q-wave MI**	8/ 2048(0.4)	7/ 2049(0.3)	1.14(0.41,3.16)	20/ 3422(0.6)	29/ 3420(0.8)	0.69(0.39,1.22)	0.02	0.39
**30-day Post-Randomization**								
**Death/MI/IDR/ST**	119/ 2044(5.8)	151/ 2040(7.4)	0.77(0.60,0.99)	207/ 3418(6.1)	229/ 3417(6.7)	0.90(0.74,1.09)	0.63	0.35
**ST**	21/ 2044(1.0)	35/ 2040(1.7)	0.59(0.34,1.03)	50/ 3418(1.5)	69/ 3417(2.0)	0.72(0.50,1.04)	0.14	0.57
**Intra-procedural ST**	8/ 2044(0.4)	16/ 2040(0.8)	0.50(0.21,1.16)	27/ 3418(0.8)	38/ 3417(1.1)	0.71(0.43,1.16)	0.04	0.48
**Definite ST**	8/ 2044(0.4)	16/ 2040(0.8)	0.50(0.21,1.16)	19/ 3418(0.6)	22/ 3417(0.6)	0.86(0.47,1.60)	0.94	0.30
**ARC ST**	14/ 2044(0.7)	20/ 2040(1.0)	0.70(0.35,1.38)	25/ 3418(0.7)	32/ 3417(0.9)	0.78(0.46,1.32)	0.99	0.80
**Death**	18/ 2044(0.9)	11/ 2040(0.5)	1.64(0.77,3.48)	42/ 3418(1.2)	44/ 3417(1.3)	0.95(0.62,1.46)	0.01	0.22
**Cardiovascular Death**	11/ 2044(0.5)	8/ 2040(0.4)	1.37(0.55,3.42)	37/ 3418(1.1)	38/ 3417(1.1)	0.97(0.62,1.53)	0.00	0.51

**MI**	81/ 2044(4.0)	125/ 2040(6.1)	0.63(0.47,0.84)	144/ 3418(4.2)	147/ 3417(4.3)	0.98(0.77,1.24)	0.06	0.02
**Q-wave MI**	3/ 2044(0.1)	7/ 2040(0.3)	0.43(0.11,1.65)	11/ 3418(0.3)	15/ 3417(0.4)	0.73(0.34,1.60)	0.23	0.50
**IDR**	24/ 2044(1.2)	30/ 2040(1.5)	0.80(0.46,1.37)	32/ 3418(0.9)	36/ 3417(1.1)	0.89(0.55,1.43)	0.12	0.77
**PCI**	18/ 2044(0.9)	26/ 2040(1.3)	0.69(0.38,1.26)	30/ 3418(0.9)	35/ 3417(1.0)	0.86(0.52,1.40)	0.52	0.58
**CABG**	6/ 2044(0.3)	4/ 2040(0.2)	1.50(0.42,5.32)	2/ 3418(0.1)	1/ 3417(0.0)	2.00(0.18,22.07)	0.00	0.83
**Death/MI/IDR/ARC-ST**	112/ 2044(5.5)	142/ 2040(7.0)	0.77(0.60,1.00)	189/ 3418(5.5)	202/ 3417(5.9)	0.93(0.76,1.14)	0.28	0.27
**Death/Q-wave MI/IDR/ST**	50/ 2044(2.4)	55/ 2040(2.7)	0.90(0.61,1.33)	98/ 3418(2.9)	117/ 3417(3.4)	0.83(0.63,1.09)	0.09	0.73
**Death/MI/IDR**	111/ 2044(5.4)	141/ 2040(6.9)	0.77(0.60,1.00)	189/ 3418(5.5)	201/ 3417(5.9)	0.94(0.76,1.15)	0.32	0.25
**Death/Q-wave MI/IDR**	42/ 2044(2.1)	41/ 2040(2.0)	1.02(0.66,1.58)	75/ 3418(2.2)	83/ 3417(2.4)	0.90(0.66,1.24)	0.34	0.64
**Death/MI/ST**	106/ 2044(5.2)	142/ 2040(7.0)	0.73(0.56,0.95)	197/ 3418(5.8)	217/ 3417(6.4)	0.90(0.74,1.10)	0.97	0.21
**Death/MI/ARC-ST**	99/ 2044(4.8)	133/ 2040(6.5)	0.73(0.56,0.95)	179/ 3418(5.2)	190/ 3417(5.6)	0.94(0.76,1.16)	0.53	0.15
**Death/Q-wave MI/ST**	35/ 2044(1.7)	43/ 2040(2.1)	0.81(0.52,1.27)	83/ 3418(2.4)	101/ 3417(3.0)	0.82(0.61,1.10)	0.01	0.97
**Death/MI**	97/ 2044(4.7)	130/ 2040(6.4)	0.73(0.56,0.96)	177/ 3418(5.2)	186/ 3417(5.4)	0.95(0.77,1.17)	0.58	0.14
**Death/Q-wave MI**	21/ 2044(1.0)	17/ 2040(0.8)	1.24(0.65,2.35)	50/ 3418(1.5)	58/ 3417(1.7)	0.86(0.59,1.26)	0.00	0.34
**Death/ST**	33/ 2044(1.6)	39/ 2040(1.9)	0.84(0.53,1.34)	77/ 3418(2.3)	95/ 3417(2.8)	0.81(0.59,1.09)	0.01	0.88

Figures are expressed as number / total number (rate). All efficacy endpoints at both time-points were assessed in patients included in the modified intention-to-treat population (which comprised patients who underwent percutaneous coronary intervention and received the study drug).

Abbreviations: ARC = Academic Research Consortium; CABG = coronary artery bypass graft surgery; CI = confidence interval; IDR = ischemia-driven revascularization; MI = myocardial infarction; OR = odds ratio; PCI = percutaneous coronary intervention; ST = stent thrombosis.

Supplemental Figure Legends

Supplemental Figure 1. Forest plot of primary efficacy endpoint assessed at 48h by individual country of enrollment. Endpoint analyses were performed in the modified intention-to-treat population (which comprised patients who underwent percutaneous coronary intervention and received the study drug). Logistic regression analyses were used to estimate effect sizes, expressed as odds ratios (OR) and 95% confidence intervals (CI).

Supplemental Figure 2. Kaplan-Meier failure curves for the key secondary efficacy endpoint, stent thrombosis, in US (**A**) and non-US (**B**) subgroups. Stent thrombosis at 48 hours after randomization was reduced by cangrelor in both US and non-US subgroups (interaction P=0.12) compared with clopidogrel in the modified intention-to-treat population (which comprised patients who underwent percutaneous coronary intervention and received the study drug). Failure functions were compared by region using the log-rank test.

Supplemental Figure 3. Kaplan-Meier failure curves for the primary safety endpoint in US (**A**) and non-US (**B**) subgroups. The primary safety endpoint, non-coronary artery bypass graft-related severe/life-threatening bleeding, according to GUSTO (Global Use of Strategies to Open Occluded Arteries) criteria at 48 h, was not significantly increased by cangrelor in both US and non-US subgroups (interaction P=0.52) compared with clopidogrel in patients who underwent randomization and received at least one dose of the study drug. Failure functions were compared by region using the log-rank test.

Supplemental Figure 1.

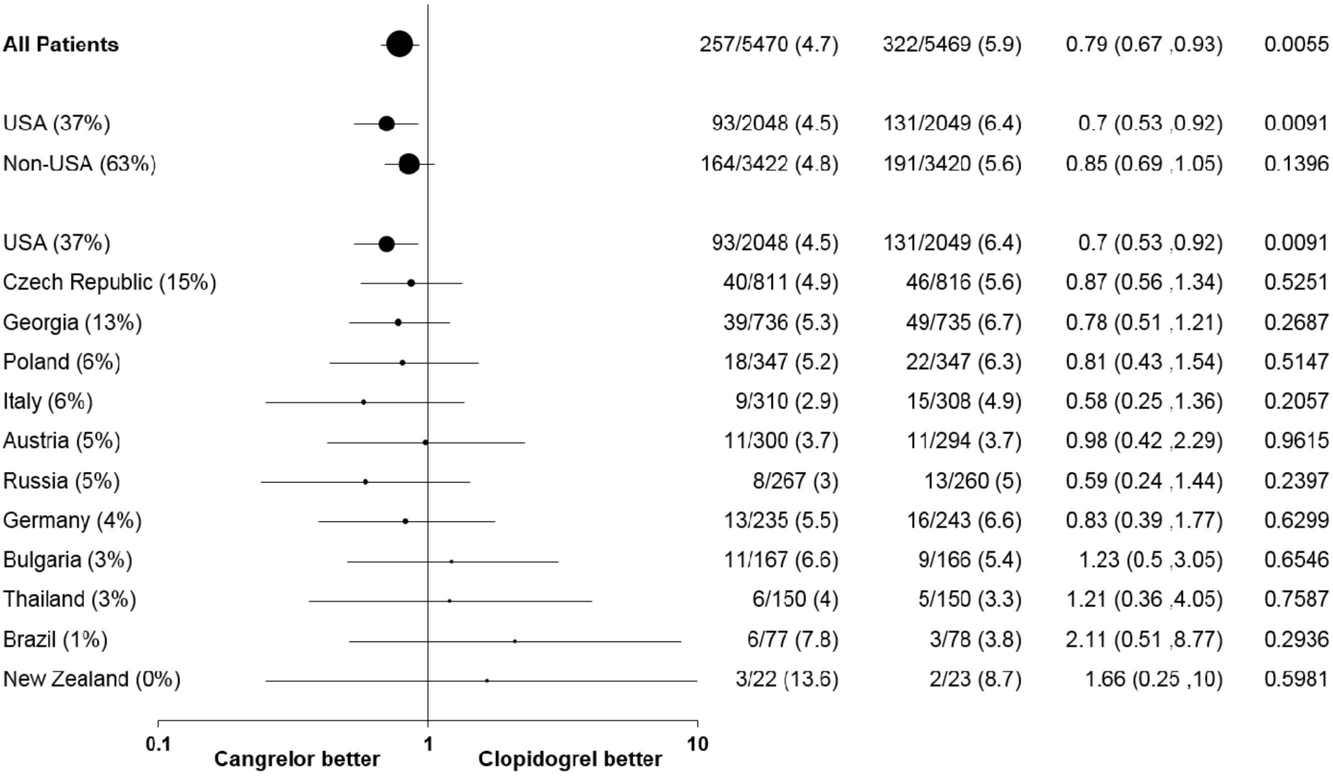

	Cangrelor	Clopidogrel	OR (95% CI)	P-value
**All Patients**	257/5470 (4.7)	322/5469 (5.9)	0.79 (0.67 ,0.93)	0.0055
USA (37%)	93/2048 (4.5)	131/2049 (6.4)	0.7 (0.53 ,0.92)	0.0091
Non-USA (63%)	164/3422 (4.8)	191/3420 (5.6)	0.85 (0.69 ,1.05)	0.1396
USA (37%)	93/2048 (4.5)	131/2049 (6.4)	0.7 (0.53 ,0.92)	0.0091
Czech Republic (15%)	40/811 (4.9)	46/816 (5.6)	0.87 (0.56 ,1.34)	0.5251
Georgia (13%)	39/736 (5.3)	49/735 (6.7)	0.78 (0.51 ,1.21)	0.2687
Poland (6%)	18/347 (5.2)	22/347 (6.3)	0.81 (0.43 ,1.54)	0.5147
Italy (6%)	9/310 (2.9)	15/308 (4.9)	0.58 (0.25 ,1.36)	0.2057
Austria (5%)	11/300 (3.7)	11/294 (3.7)	0.98 (0.42 ,2.29)	0.9615
Russia (5%)	8/267 (3)	13/260 (5)	0.59 (0.24 ,1.44)	0.2397
Germany (4%)	13/235 (5.5)	16/243 (6.6)	0.83 (0.39 ,1.77)	0.6299
Bulgaria (3%)	11/167 (6.6)	9/166 (5.4)	1.23 (0.5 ,3.05)	0.6546
Thailand (3%)	6/150 (4)	5/150 (3.3)	1.21 (0.36 ,4.05)	0.7587
Brazil (1%)	6/77 (7.8)	3/78 (3.8)	2.11 (0.51 ,8.77)	0.2936
New Zealand (0%)	3/22 (13.6)	2/23 (8.7)	1.66 (0.25 ,10)	0.5981

Supplemental Figure 2A.

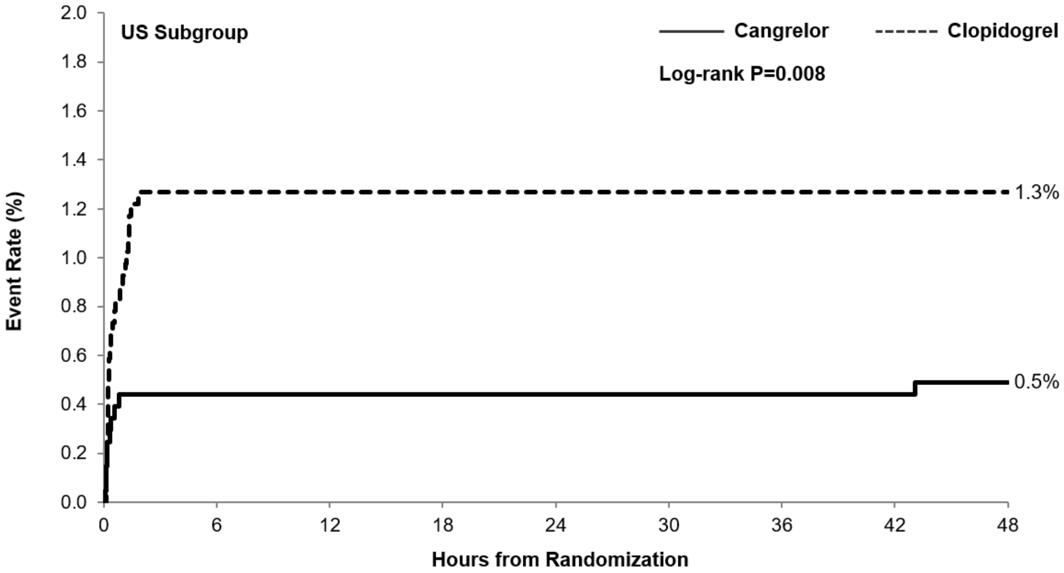

Patients at Risk									
Cangrelor	2048	2037	2036	2036	2036	2035	2035	2035	2034
Clopidogrel	2049	2023	2023	2023	2023	2023	2023	2023	2023

Supplemental Figure 2B.

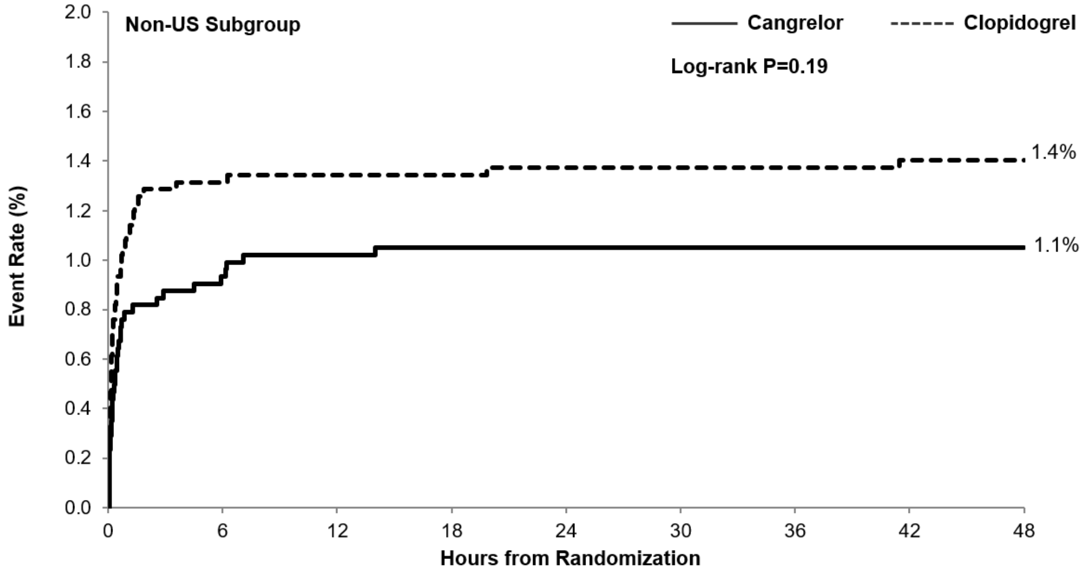

Patients at Risk									
Cangrelor	3424	3389	3385	3383	3383	3383	3382	3381	3380
Clopidogrel	3421	3369	3366	3365	3363	3362	3362	3360	3360

Supplemental Figure 3A.

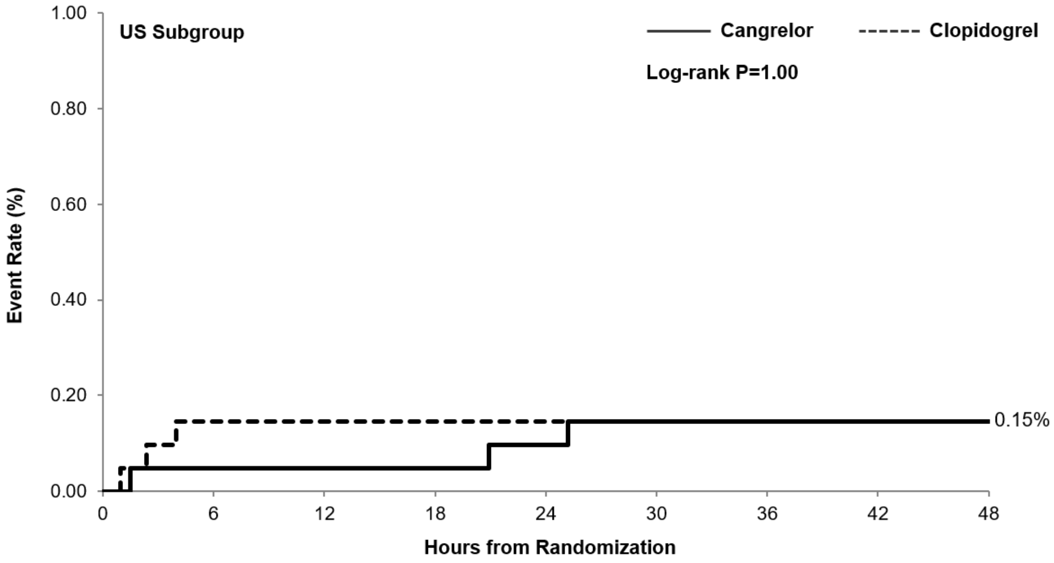

Patients at Risk									
Cangrelor	2062	2058	2057	2057	2056	2054	2054	2054	2054
Clopidogrel	2062	2059	2059	2059	2059	2059	2059	2059	2059

Supplemental Figure 3B.

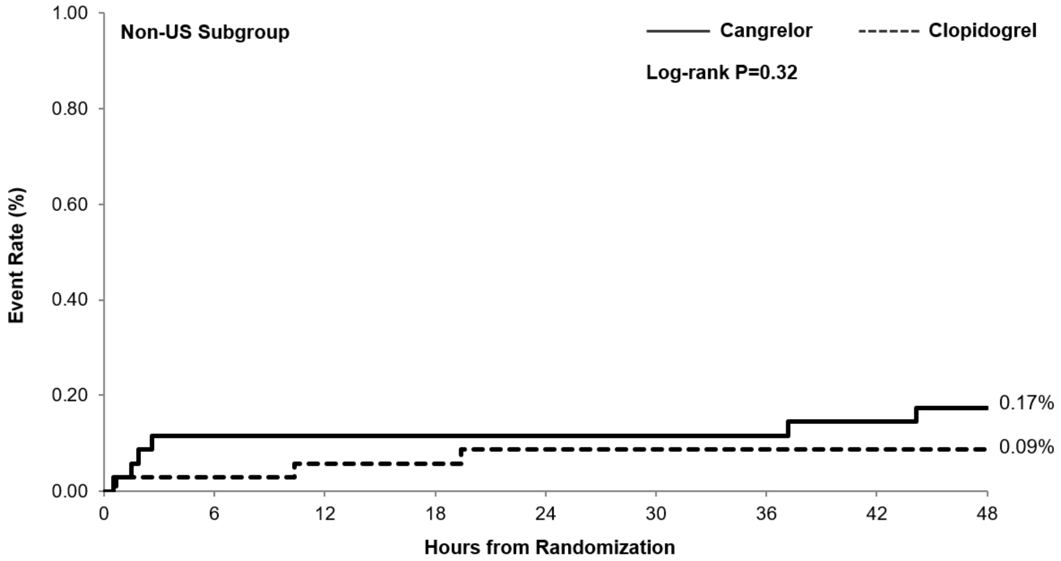

Patients at Risk									
Cangrelor	3467	3458	3456	3455	3455	3455	3454	3451	3447
Clopidogrel	3465	3452	3449	3448	3446	3443	3443	3441	3441
